# Mechanisms of False Alarm in Response to Fear Stimulus: An Event-Related Potential Study

**DOI:** 10.3389/fnhum.2021.730011

**Published:** 2022-01-28

**Authors:** Xiai Wang, Jicheng Sun, Jinghua Yang, Shan Cheng, Cui Liu, Wendong Hu, Jin Ma

**Affiliations:** ^1^School of Aerospace Medicine, Air Force Medical University, Xi’an, China; ^2^Officers College of PAP, Chengdu, China; ^3^Department of Research and Academic, Air Force Medical University, Xi’an, China; ^4^Fundamentals Department, Air Force Engineering University, Xi’an, China

**Keywords:** ERP, False Alarm (FA), fear stimuli, oddball paradigm, snake

## Abstract

**Background and Objective:**

There is a paucity of research that has explored “False Alarm” mechanisms. In order to remedy this deficiency in knowledge, the present study used event-related potential (ERP) technology to reveal the mechanisms underlying False Alarm in response to fear stimuli.

**Methods:**

This study selected snakes as experimental materials and the “oddball paradigm” was used to simulate the conditions of False Alarm. The mechanism underlying False Alarm was revealed by comparing cognitive processing similarities and differences between real snakes and toy snakes.

**Results:**

Event-related potential findings demonstrated that there was no significant difference between N1 and P2 components induced by real and toy snakes in the early processing stage. Compared with toy snakes, real snakes induced smaller N2 amplitude, larger P3 amplitude, and a shorter P3 latency at the late processing stage. The results of brain topographic mapping analysis showed that the brain regions activated by a real or toy snake were basically the same within the time windows of 110–150 and 220–270 ms, respectively. In the time window of 300–360 and 400–500 ms, the degree of brain regions activation with a real snake was significantly greater than that induced by a toy snake.

**Conclusion:**

False Alarm is caused by the brain’s inability to distinguish, in the early stage of cognitive processing, stimulus objects with similar appearances. When the brain is able to distinguish the differences between different stimulus objects in the late stage of cognitive processing, False Alarm disappears.

## Introduction

The concept of “False Alarm” in psychology comes from the theory of signal detection, which is defined as the reporting of the specified signal when there is no specified signal (Landry et al., 2021), while the “False Alarm” in response to the fear stimulus refers to the fear response to the stimulus, which is similar to the fear stimulus but does not indicate a substantial threat (LoBue, 2014). For example, when you are walking and suddenly find a “snake” in the grass by the side of the road, you will instantly have a strong sense of fear, but as soon as you find out that it is a toy snake, the fear will rapidly subside; this is a False Alarm.

After a literature search, it is clear that there is a paucity of psychological studies on False Alarm. For example, Reinhard et al. (2020) believed that the False Alarm of fear had the same function as the generalization of fear, both of which can enhance the alertness of an individual to better deal with threatening stimuli. Lissek et al. (2010, 2014) believed that patients with a generalized anxiety disorder, panic disorder, specific phobias, or other psychological diseases had a significantly higher probability of False Alarm than healthy individuals. Mowen et al. (2004) found that False Alarm affected an individual’s attention to visual cues. Studies related to personality have also reported that people with sensitive personality traits were more prone to a False Alarm reaction (Brand et al., 2016; Tay, 2018; De Pascalis et al., 2019), and both real fear and False Alarm can cause a startle reflex (Anokhin, 2017; Dhamija et al., 2017; Savage et al., 2019), etc. Unfortunately, few mechanisms have been reported for the generation of False Alarm. It is of great significance to reveal the mechanisms underlying False Alarm, which will not only expand on the depth and breadth of fear research, but also deepen the understanding of fear generalization, and provide a theoretical basis for the treatment of fear-related disorders.

Thus, the present study explored the time process of False Alarm with event-related potential (ERP) technology to identify the neural mechanisms underlying False Alarm. False Alarm usually appears when people suddenly encounter a fear stimulus, with obvious “sudden” characteristics. In order to improve the ecological validity of the research, our study used the Oddball paradigm to present stimuli. In the Oddball paradigm, a stimulus is presented in a completely random manner in which the probability of the standard stimulus is ≥70%, and the probability of each non-standard stimulus is ≤10% (van Dinteren et al., 2017). As the occurrence probability of different types of stimuli varies greatly, when non-standard stimuli appear in a completely random manner they are equivalent to a sudden change in the background constituted by the standard stimuli. Therefore, the sudden occurrence of False Alarm can be well simulated.

## Materials and Methods

### Design

A single-factor within-subject design was adopted, the independent variable was the type of stimulus, and the dependent variables were the amplitude and latency of N1, P2, N2, and P3. Studies have proven that snakes were a typical representation of fear (Isbell, 2006; Erlich et al., 2013; Coelho et al., 2021). This study used previous research methods to induce fear by taking pictures of snakes (Langeslag and van Strien, 2018; Masataka et al., 2018; Beligiannis and Van Strien, 2019; Bertels et al., 2020). In view of the fact that the object of False Alarm must be “similar to the real fear object but not physically threatening,” toy snakes similar to a real snake were selected as the object of False Alarm, and the mechanisms underlying False Alarm were revealed by comparing cognitive processing similarities and differences between the two types of stimuli. Toy snakes were chosen to look like real ones, but to be recognizable as toys. At the same time, in order to enhance contrast, our study introduced neutral stimuli such as household items as the control and three types of stimuli: a real snake, toy snake, and a neutral stimulus. According to the characteristics of False Alarm, it is hypothesized that in the early processing stage, the brain has similar processing characteristics for a real snake and the toy snake, while in the late processing stage, the real snake and the toy snake produce significant differences in cognitive processing.

### Participants

This study was approved by the ethics committee of the First Affiliated Hospital of Fourth Military Medical University. All participants in these experiments were college students, who were screened for potential panic disorder, specific phobias, post-traumatic stress disorder (PTSD), generalized anxiety disorder, and other fear-related disorders using questionnaires. Only those who answered “No” to these questions were selected to take part in the formal experiments. A nine-point Likert scale was adopted to evaluate the fear sensitivity of each participant and eliminated those with excessively low (score ≤ 3) or high (score ≥ 7) fear sensitivity scores. A total of 24 participants were recruited for the experiments, such as 1 with excessively high fear sensitivity, 2 with excessively low fear sensitivity, and 1 with data recording problems. These participants were excluded as invalid participants, and finally, 20 effective participants (12 men, 8 women) were enrolled, aged 20–24 years (21.8 ± 1.72). All of them were right-handed, with normal or vision that had been optically corrected and did not have a history of brain injury or disease. Informed consent forms were signed by all participants before the experiments began. After completing all the experimental tasks, each participant was paid 100 RMB.

### Stimuli

**FIGURE 1:**
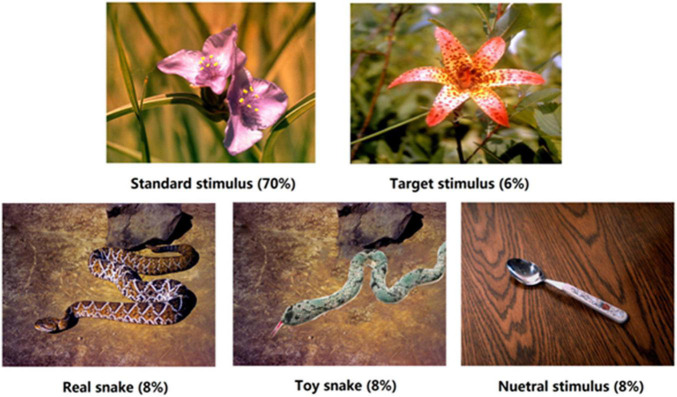
Schematic diagrams of the five types of stimuli. Each block contained 200 trials in total, such as standard stimulus 140 times (70%), target stimulus 12 times (6%), and each type of bias stimulus 16 times (8%), respectively.

The experimental materials were obtained from the International Affective Picture System (Lang et al., 2001), and were divided into five types of stimuli. The standard stimulus was a flower image, and the target stimulus was another flower image. Bias stimuli were a real snake, toy snake, and neutral images, with 8 images of each bias stimulus (see Figure 1). The toy snake image was modified from the real snake image, mainly using Photoshop (PS) software to replace the real snake with a similar toy snake; other elements remained the same. PS technology was used to adjust the brightness and size of each image to ensure that their physical properties were consistent. In order to eliminate the contamination due to material familiarity of the experimental results, participants were asked to evaluate the familiarity of the experimental materials. ANOVA showed that there was no significant difference in the familiarity of the three types of stimuli, *F*_(2_,_38)_ = 1.05, *p* > 0.05. What is worth to be mentioned is that in the brain cognition study of fear, it is enough to make the subjects feel the threat of fear, and it is not necessary to bring a substantial threat to the participants. The real snake pictures in this study could make the subjects fear threat, while the toy snake pictures did not. Therefore, using picture materials can simulate the conditions for False Alarm well.

### Procedures

**FIGURE 2:**
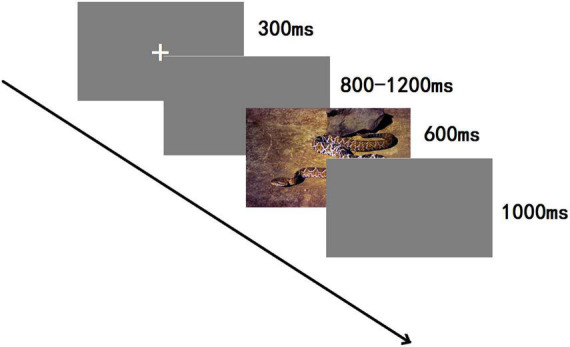
The sequence of events in an experimental trial.

A 3-stimulus passive oddball paradigm was employed (see Figure 2), and stimuli were randomly presented using E-Prime 3.0. The whole experiment contained 800 trials in total, divided into four blocks. A 3 min rest was allowed between each block. In each block, the standard stimulus was shown 140 times (70%), the target stimulus 12 times (6%), and each type of bias stimulus 16 times (8%), respectively; each image of the bias stimulus was presented two times in one block. Participants were seated comfortably in a quiet room about 80 cm from the computer screen; the visual vertical and horizontal angles were <5 degrees. Each experiment was begun by showing, for 300 ms, a small white cross on a blank screen and then on a computer screen, which was randomly altered from 800 to 1,200 ms duration. Then, one of the five categories of stimuli was displayed for 600 ms. After the presentation of a visual stimulus, a blank screen was shown for 1,000 ms. The participants had to carefully observe and initiate a behavioral response only to target stimuli within the shortest possible time. No responses were needed for a standard stimulus or the three categories of emotional images. To hide the real nature of the investigations, a participant was informed that it was a reaction time experiment, and the results of reaction times were given after completing the experiment.

### Data Recording and Analysis

A total of 64 scalp electrodes were positioned following the international 10–20 system to measure the electroencephalograms (EEGs), which were continuously recorded. All electrodes were referenced on-line to one positioned on the left mastoid and re-referenced off-line to one placed on the bilateral mastoid. The horizontal electrooculogram (EOG) was recorded using 2 electrodes placed 1.5 cm lateral to the left and right outer canthi and the vertical EOG from 2 electrodes placed above and below the left eye (impedance < 5 kΩ). The amplified EEG was digitized at 1,000 Hz and for each stimulus type was analyzed off-line with averaging epochs than commenced 200 ms prior to the stimulus onset and ended 600 ms after the onset. Trials affected by blinking of the eyes (VEOG more than ±50 μV) or other artifacts (more than ±50 μV at any electrode site) relative to baseline were considered to be contaminated and excluded from the dataset. Based on previous research methods (Fan et al., 2016), fifteen electrodes were selected for the statistical analyses, namely, F3, FC3, C3, CP3, P3 (left placements); Fz, FCz, Cz, CPz, Pz (midline placements); and F4, FC4, C4, CP4, P4 (right placements). A three-factor repetition measures ANOVA was carried out on the latency and amplitude of N1, P2, N2, and P3. The matching ANOVA variables were stimulus type (real snake, toy snake, and neutral), frontality (front, front-central, central, central-parietal, and parietal sites), and laterality (left, middle, and right sites). The degrees of freedom for the *F*-ratio were corrected by the Greenhouse–Geisser method. In addition, brain topographic mapping analysis of N1, P2, N2, and P3 components was conducted to explore the similarities and differences of brain regions activated by real and toy snakes.

## Results

### Event-Related Potential Component Analysis

**FIGURE 3:**
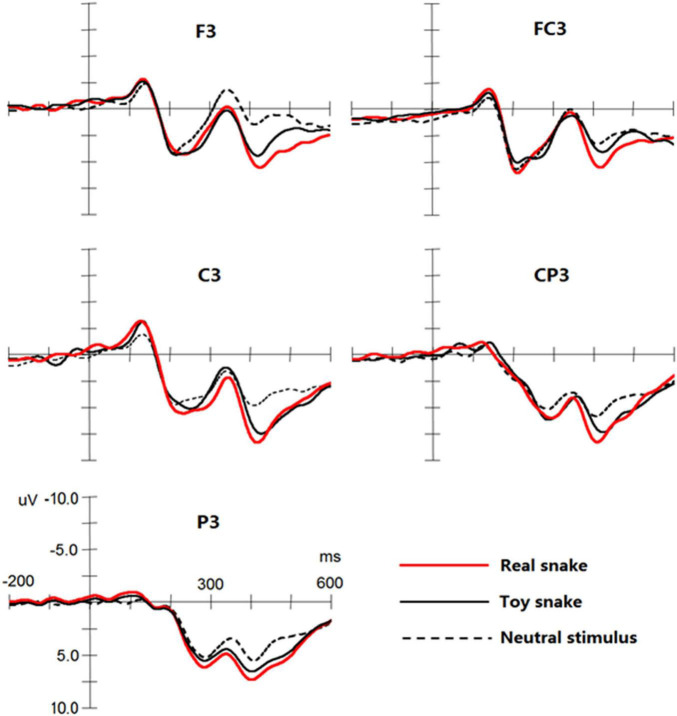
Event-related potential (ERP) components elicited by a real snake, toy snake, and neutral stimuli at the F3, FC3, C3, CP3, and P3 electrodes.

The results showed that significant N1, P2, N2, and P3 components were elicited by the three types of stimuli (see Figure 3), and three-way repeated measures of analyses of variance were conducted for the amplitude and latency of each component.

#### N1 Component

In the latency of N1, the main effect and interaction effect of stimulus type were not significant. On the N1 amplitude, the interaction between stimulus type and frontality was significant, *F*_(8_,_152)_ = 3.42, *p* < 0.05, ω^2^ = 0.12, simple effect analysis found that neutral stimulation in the central sites [*F*_(1_,_19)_ = 2.33, *p* < 0.05] and central-parietal sites [*F*_(1_,_19)_ = 1.99, *p* < 0.05] elicited N1 amplitude was significantly lower than that of the real and toy snakes, but there was no significant difference between real and toy snakes.

#### P2 Component

The main effect and interaction effect of stimulus type were not significant on P2 latency. On the P2 amplitude, the interaction between the stimulus type and the frontality was significant, *F*_(8_,_152)_ = 3.87, *p* < 0.05, ω^2^ = 0.14, simple effect analysis found that real and toy snake; the P2 amplitudes elicited in the central [*F*_(1_,_19)_ = 2.41, *p* < 0.05] and parietal [*F*_(1_,_19)_ = 2.29, *p* < 0.05] sites were significantly greater than those elicited by neutral stimuli, but there was no significant difference between real and toy snakes.

#### N2 Component

The main effect and interaction effect of the stimulus type were not significant during the N2 latency. On the N2 amplitude, the interaction between the stimulus type and the frontality was significant, *F*_(8_,_152)_ = 5.94, *p* < 0.05, ω^2^ = 0.20. Simple effect analysis showed that the amplitude of N2 elicited by the real snake was significantly smaller than that elicited by the toy snake and neutral stimuli in the central [*F*_(1_,_19)_ = 3.28, *p* < 0.05] and parietal [*F*_(1_,_19)_ = 3.28, *p* < 0.05] sites. The interaction between stimulus type and laterality was significant [*F*_(4_,_76)_ = 5.29, *p* < 0.05, ω^2^ = 0.17]. Simple effect analysis revealed that the N2 amplitude elicited by real snakes in the left brain was significantly lower than that of the right brain [*F*_(1_,_19)_ = 4.07, *p* < 0.05] and midbrain [*F*_(1_,_19)_ = 3.88, *p* < 0.05]; there was no significant difference between the right brain and midbrain.

#### P3 Component

The interaction between the stimulus type and the frontality was significant in the P3 latency, *F*_(8_,_152)_ = 5.29, *p* < 0.05, ω^2^ = 0.22, simple effect analysis showed that the latency of P3 elicited by real snakes were significantly shorter than that of toy snakes in the front-central sites [*F*_(1_,_19)_ = 1.87, *p* < 0.05], central sites [*F*_(1_,_19)_ = 2.32, *p* < 0.05], and central-parietal sites [*F*_(1_,_19)_ = 2.38, *p* < 0.05]. On the P3 amplitude, the main effect of stimulus type was significant, *F*_(2_,_38)_ = 14.55, *p* < 0.01, ω^2^ = 0.24, the amplitude elicited by real snake was greater than that of toy snake [*F*_(1_,_19)_ = 2.76, *p* < 0.05] and neutral stimulus [*F*_(1_,_19)_ = 4.67, *p* < 0.05], the amplitude elicited by toy snake was greater than that of neutral stimulus [*F*_(1_,_19)_ = 4.12, *p* < 0.05]. The interaction between stimulus type and frontality was significant, *F*_(8_,_152)_ = 10.29, *p* < 0.05, ω^2^ = 0.25. Simple effect analysis demonstrated that the P3 amplitude elicited by a real snake was greater than that of the toy snake and neutral stimulus at all electrodes [all *F*s_(1_,_19)_ > 1.73, all *p*s < 0.05]. The interaction between the stimulus type and laterality was significant, *F*_(4_,_76)_ = 5.21, *p* < 0.05, ω^2^ = 0.16. Simple effect analysis showed that the amplitude elicited by real snakes in the left brain was significantly higher than that in the midbrain [*F*_(1_,_19)_ = 2.57, *p* < 0.05] and right brain [*F*_(1_,_19)_ = 2.81, *p* < 0.05]; there was no significant difference between the midbrain and right brain.

### Brain Topographic Mapping Analysis

**FIGURE 4:**
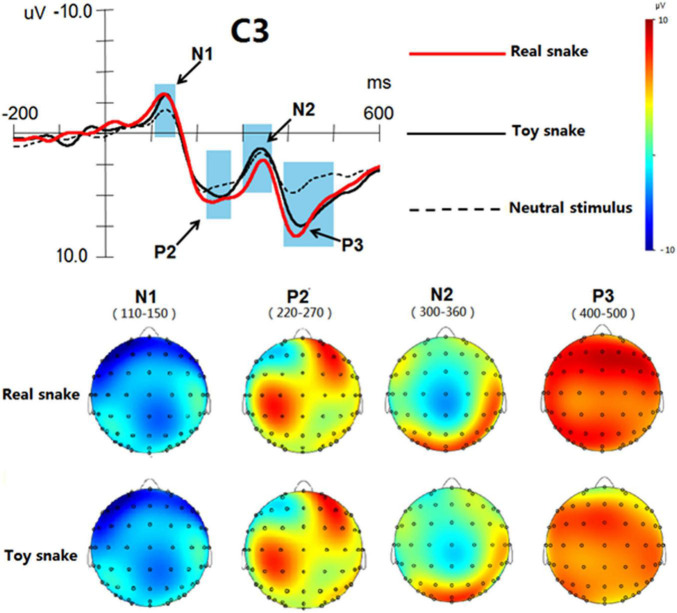
Brain topographic maps of ERP components induced by real and toy snake.

In the Analyzer software, the time window of each ERP component was determined according to the butterfly diagram. The time window of N1 was 110–150 ms, the time window of P2 220–270 ms, the time window of N2 300–360 ms and the time window of P3 400–500 ms. Among the electrodes investigated, the difference at the C3 recording electrode was the most obvious, so the C3 electrode was selected to construct the brain topographic maps of each ERP component (as shown in Figure 4). In terms of the regions, the activated regions of real and toy snakes were similar in the same time window. In terms of the degree of activation, the activation degree of real and toy snakes was basically the same in the time windows 110–150 and 220–270 ms, while the activation degree of real snakes was significantly greater than that of toy snakes in the time windows of 300–360 and 400–500 ms.

## Discussion

The results of ERP analysis showed that N1, P2, N2, and P3 waveforms were elicited by three types of stimuli. The N1 and P2 amplitudes elicited by real and toy snakes were significantly greater than those elicited by neutral stimuli, indicating that fear stimuli were more likely to capture early attention than neutral stimuli, a finding consistent with previous studies (Grassini et al., 2018; Jing et al., 2019). It is worth noting that in this study, no significant difference was found in N1 and P2 composition between real and toy snakes. N1 and P2 components usually represent the initial perception of brain and rapid detection of the stimulus object (Wei et al., 2016; Qun et al., 2018), and are early processing indicators of the brain (Zhou et al., 2017; Heacock et al., 2019). The physical appearance of real and toy snakes is very similar, and they elicited almost the same N1 and P2 amplitudes, indicating that the brain has the same preliminary perception of real and toy snakes, and the detection speed is basically the same. The results may well explain the emergence of False Alarm: the brain’s cognitive processing of stimuli in the early stages is crude and superficial, and usually does not involve the allocation of cognitive resources, and the brain cannot tell the difference between real and toy snakes when they have a similar appearance. In other words, when the False Alarm object is physically similar to the real threatening stimulus, the brain receives almost exactly the same information at an early stage, so the False Alarm object will be perceived as the real threatening stimulus, thus leading to the emergence of False Alarm.

After the N1 and P2 components were elicited, the stimulus images continued to elicit N2 and P3 components. N2 and P3 are present in the later stages of cognitive processing and are typically involved in the allocation of cognitive resources, when the brain is more refined in processing the stimulus object (Hamilton et al., 2019; Schlüter et al., 2019; Silva-Sauer et al., 2019). The results showed that there were significant differences between the toy and real snakes at this stage, indicating that the brain has been carefully prepared to distinguish the differences between them. In the Oddball paradigm of visual tasks, N2 is a non-specific component, which is usually associated with the attention switching mechanism (Buodo et al., 2015; Citherlet et al., 2019; Schlüter et al., 2019). The N2 amplitudes elicited by the real snake in the central and parietal sites were significantly smaller than that elicited by a toy snake, indicating that the real snake consumes less cognitive resources during the attention switching process, and the switching is easier. The P3 component represents the brain’s deep processing of the stimulus object, which belongs to the most delicate processing stage (Wei et al., 2016; Qun et al., 2018). The shorter the latency period, the earlier the processing, the greater the amplitude, and the more cognitive resources attracted (Hamilton et al., 2019; Schlüter et al., 2019; Silva-Sauer et al., 2019). It can be seen from the results that the P3 amplitude elicited by the real snake was significantly greater than that produced by the toy snake, and the latency was significantly shorter than for the toy snake, indicating that the brain allocates more cognitive resources to the real snake, and the processing degree is deeper and the processing time earlier. Thus, the difference in N2 and P3 was enough to show that the brain was already able to distinguish between the real and toy snake.

At the same time, the familiarity of the material was controlled and the influence of irrelevant factors excluded, which further indicated that the differences in the results reflected differences between the real and toy snake in cognitive processing. In terms of the time course of cognitive processing, N2 and P3 components appear at 300–500 ms within the time window, that is, the brain can discern the perceived object is a real threat or not when the stimulus rendering is 300–500 ms. When the brain determines that the perceived target is not threatening, the False Alarm will lose the basis for its generation, and it will quickly disappear. This may explain why the False Alarm is so brief.

The results of brain topographic mapping analysis showed that the brain regions activated by the real snake and the toy snake were basically the same at both the early and late processing stages, suggesting that the two types of stimuli had similar processing characteristics during cognition. However, in terms of the degree of activation, there was no significant difference between the real snake and the toy snake at the early stage, but there was a significant difference at the late stage, indicating that the brain could only distinguish the difference between the two at the late processing stage. This result further verified the results of ERP analysis. From the perspective of brain regions, the ERP components elicited by real and toy snakes exhibited the most significant differences in the left brain, indicating that related brain structures in the left brain played a decisive role in the process of identifying whether the perceived object was truly threatening.

In this study, ERP technology was used to reveal the neural mechanism of False Alarm. Compared with other technologies, the biggest advantage of ERP lies in its high temporal resolution (accurate to 1 ms), which can accurately reflect the time course of brain cognitive processing, but it also has the inherent defect of low spatial resolution (Wei et al., 2016). Thus, it is not clear which brain structures are activated during False Alarm. In this sense, the understanding of the mechanism underlying False Alarm in this study is not comprehensive enough. In future research, high spatial resolution techniques such as functional MRI (fMRI) technology will be adopted to remedy this deficiency. Furthermore, fear can be expressed in a variety of forms, according to the difference in nature; it can be roughly divided into “evolutionary fear” and “modern fear,” among which evolutionary fear is represented by snakes and modern fear by guns (Dhum et al., 2017; Subra et al., 2018; LoBue and Adolph, 2019). In the present study, snakes were chosen as the study subjects to examine only the False Alarm of evolutionary fear. Studies have proven that evolutionary fear and modern fear have essential differences, they have different origins and that their cognitive processing patterns are also significantly different (Erlich et al., 2013; Subra et al., 2018; Ren and Tao, 2020). Therefore, it can be inferred that the emergence of False Alarm of modern fear is likely to have a mechanism or characteristics different from that of evolutionary fear. In further research, guns will be used as stimulus materials to focus on the mechanism of False Alarm in response to modern fear stimuli, so as to build a more complete understanding of the mechanisms underlying False Alarm.

## Conclusion

The experiments analyzed the time course of False Alarm using high temporal resolution ERP technology and identified the potential neural mechanisms of False Alarm. False Alarm is caused by the inability of brain to distinguish, in the early stage of cognitive processing, stimulus objects with similar appearances. When the brain is able to distinguish the differences between different stimulus objects in the late stage of cognitive processing, False Alarm disappears.

## Data Availability Statement

The datasets presented in this study can be found in online repositories. The names of the repository/repositories and accession number(s) can be found in the article/supplementary material.

## Ethics Statement

The studies involving human participants were reviewed and approved by Ethics Committee of the First Affiliated Hospital of Fourth Military Medical University. The patients/participants provided their written informed consent to participate in this study.

## Author Contributions

XW, WH, and JM conceived and designed the experiments. XW and JY performed the experiments. XW, JY, and SC analyzed the data. JS and CL were responsible for the resources and software. XW, JY, and JS wrote the original draft. All authors contributed to the article and approved the submitted version.

## Conflict of Interest

The authors declare that the research was conducted in the absence of any commercial or financial relationships that could be construed as a potential conflict of interest.

## Publisher’s Note

All claims expressed in this article are solely those of the authors and do not necessarily represent those of their affiliated organizations, or those of the publisher, the editors and the reviewers. Any product that may be evaluated in this article, or claim that may be made by its manufacturer, is not guaranteed or endorsed by the publisher.
